# THE PAID AND FAMILY DEMENTIA WORKFORCE IN THE COVID-19 PANDEMIC ERA

**DOI:** 10.1093/geroni/igad104.1767

**Published:** 2023-12-21

**Authors:** Karen Donelan

## Abstract

The COVID-19 pandemic has had a disproportionate impact on persons living with dementia and their carers. The early period of the pandemic led to drastic changes in dementia care at home, in healthcare services, community programs, and institutional care settings, with lasting effects (Capstick et al., 2022; Giebel et al., 2022). This symposium describes the impact of the COVID-19 pandemic on a variety of individuals providing care for persons living with dementia through different stages of the pandemic. Our work will be presented within a translational framework and include a discussion of ways of leveraging findings to improve resources for individuals and caregivers living with dementia in the COVID-19 era. Karen Donelan will provide an overview of the dementia workforce and describe study procedures. Michael Vetter will describe findings from a survey study of self-efficacy of dementia caregiving for family caregivers of individuals in community and assisted living facilities (ALF). Inga Antonsdottir will describe survey data on family caregivers in community and ALF – focus on newer experiential members; how lives changed, how COVID impacted them. Esteban Barreto will present findings describing the impact of the COVID-19 pandemic on frontline staff. Finally, Sarah Bannon will present findings from a qualitative study identifying family caregivers’ impressions of their psychosocial stressors and enacted coping strategies in the post-vaccine era of the COVID-19 pandemic.

